# Keeping track of all ongoing colorectal cancer trials using a mobile application: Usability and satisfaction results of the Dutch Colorectal Cancer Group Trials application

**Published:** 2018-12-16

**Authors:** Joost Huiskens, Michael S. Gałek-Aldridge, Jean-Michel Bakker, Pim B. Olthof, Thomas M. van Gulik, Cornelis J. A. Punt, Martijn G. H. van Oijen

**Affiliations:** ^1^Department of Surgery, Academic Medical Center, Amsterdam, Netherlands; ^2^Department of Medical Oncology, Academic Medical Center, Amsterdam, Netherlands; ^3^Department of Reinier de Graaf Gasthuis, Delft, Amsterdam, Netherlands

**Keywords:** clinical trials, colorectal cancer, smartphone application

## Abstract

**Background and Aim:**

Both the number and complexity of medical trials are increasing vastly. To facilitate easy access to concise trial information, a freely available mobile application including all ongoing clinical trials of the Dutch Colorectal Cancer Group (DCCG) was developed. The aim of this study was to investigate the use and user satisfaction over the first 2 years.

**Methods:**

The application was launched in January 2015 on iOS and Android platforms. Google Analytics was used to monitor anonymous user data up to February 2017. In addition, an online survey regarding the use and satisfaction among health-care professionals and research affiliates active in the field of colorectal cancer in the Netherlands was conducted.

**Results:**

A total of 6173 unique users were identified, of which 1822 (30%) were from the Netherlands, representing a total of 16,065 and 10,987 (68%) sessions, respectively. The median session duration per day was 01:47 min (IQR 0:51–03:03). The mobile application was mostly used on Monday, Tuesday, and Thursday, and the number of sessions was highest during the following time frames: 12–13 pm (9%), 17–18 pm (9%), and 13–14 pm (8%). Of 121 survey responses, most were medical doctors (47%), nurses (25%), or researchers (9%), working either in a teaching (40%), academic hospital (32%), or general hospital (19%). 83% of all respondents rated the application 4 or higher for satisfaction on a 5-point scale. Highest reported reasons of the use were urgent trial inquiry (57%) and usage during multi-disciplinary meetings (49%).

**Conclusion:**

The DCCG Trials application is frequently used, and the majority of users is highly satisfied.

**Relevance for Patients:**

Clustering trial information into one platform, such as DCCG trials app, has shown to be useful for medical professionals treating patients with colorectal carcinoma in the Netherlands.

## 1. Introduction

**Figure 1:**
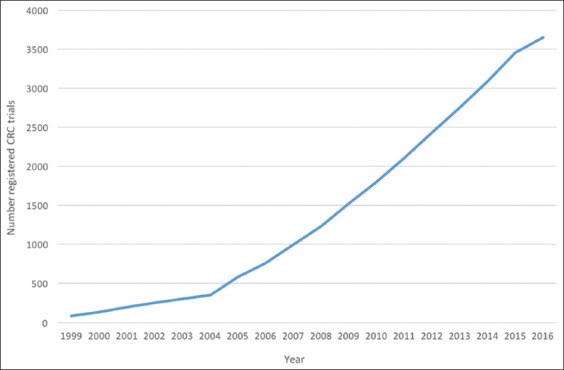
Number of registered trials on colorectal carcinoma in clinicaltrials.gov.

The number of registered clinical trial protocols on clinicaltrials.gov has increased from 12,020 in 2005 to over 230,000 in 2017, and the yearly number of newly registered studies is approaching 30,000 [1]. In the field of colorectal cancer alone, the third most common cancer worldwide, 4482 trials, was registered by the end of 2017 (Figure 1).

Besides the increasing number of clinical trials, there has been an increase in protocol design complexity during the past decade. The incidence of added trial protocol amendments is growing and trial criteria are increasingly specified and complex [2]. Among many others, these factors lead to the shortage of both knowledge and time for health-care professionals to participate in clinical trials [3,4]. The complexity of clinical trials potentially hampers the inclusion of patients, which is the leading cause of problems in the conduction of clinical trials [5-8]. Lack of accrual can lead to trial discontinuation or results in an insufficient sample size, both of which challenge the ethics of the exposure of patients to trial treatment which is often outside of routine clinical practice.

Evidence on effective strategies to improve the conduction of clinical trials is scarce and mostly limited to the recruitment for randomized trials [9]. The Cochrane systematic review of interventions to improve the trial recruitment included 45 studies, of which only 12 were considered to be of low risk of bias [10]. Opt-out procedures, telephone reminders, and open trial designs might be effective strategies to improve patient recruitment. However, these strategies do not comply with the guidelines of good clinical practice. Interestingly, none of these reported interventions targeted medical doctors, who usually assess patient eligibility for trials and ask patients to participate in clinical trials.

This manuscript illustrates the design of a smartphone application that provides easy to access and up-to-date information on ongoing Dutch clinical trials for patients with colorectal cancer. The aim of this study is to investigate the usability and satisfaction of the application 2 years after its introduction.

## 2. Materials and Methods

Anonymous user data of the Dutch Colorectal Cancer Group (DCCG) Trials application were collected between the February 1 2015 and February 1 2017 and analyzed using Google Analytics [11]. In addition, an online survey among health-care professionals and research affiliates active in the field of colorectal cancer was conducted.

**Figure 2:**
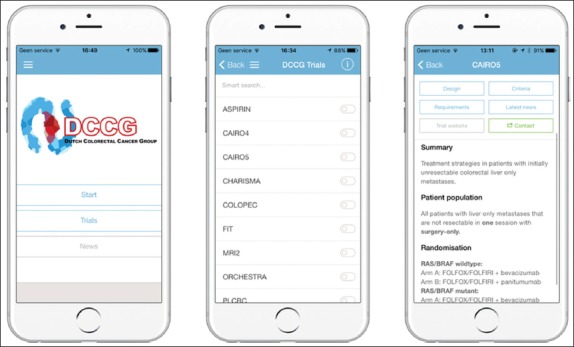
Illustration of the home screen, trials overview, and a trial information page.

The DCCG is a research collaboration in the Netherlands between all medical disciplines involved in the diagnosis and management of colorectal cancer [12]. The DCCG Trials application is a mobile application containing concise information on all the DCCG multicenter trials open for inclusion. The application is freely downloadable for iOS and Android [13]. It can also be accessed through the website: www.trialapp.nl/dccg/dccg-app/without using the mobile application. Users can find relevant information for the registration of a patient for a trial. Trial coordinators can be e-mailed or called directly through the application for questions. The information provided in the application per trial can be directly updated by the responsible investigators. Trials can be found in two different ways, either by following a decision-tree based on clinical features or by utilizing the search function. Using the decision-tree users can search for trials by answering questions such as; is the tumor located in the colon, rectum, or does the patient have metastatic disease? Every trial page has six buttons: Design, criteria, requirements, latest news, the trial website, and contact information (Figure 2).

Anonymously collected user data by Google Analytics [11] included the number of (new) users, the number of sessions, the bounce rate (users that opened and closed the app immediately), average session duration per user per day, geographical location based on IP address, the number and duration of trial information page visits, the number of times the decision-tree option which was used, and the amount the news option which was used.

To measure usability and satisfaction, an online survey was sent either through the DCCG Trials application, in the DCCG newsletter, or by email using the DCCG mailing list, consisting of email addresses of health-care professionals and research affiliates. The survey included 10 questions regarding the respondent’s profession, usability, and satisfaction of the DCCG Trials application, using a 5-point Likert scale. An overview of the online survey is shown in Appendix 1.

## 3. Results

**Supplementary Figure 1:**
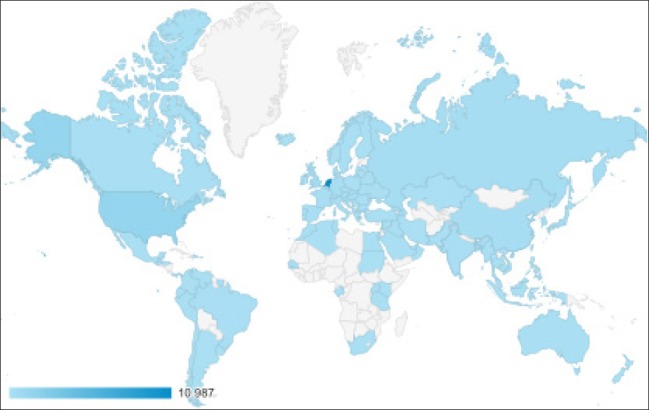
Overview of the reasons for application use. 75 of 121 people answered this question.

In its first 2 years, the DCCG Trials application amassed a total of 16,065 sessions and 89,711 page views by 6,173 unique users worldwide. The median session duration per day was 01:47 min (IQR 0:51–03:03). The median number of pages visited per session was 6 (IQR 4–7). In total, the application was used in 102 countries (Supplementary Figure 1).

In the Netherlands, 1,822 (30%) unique users undertook a total of 10,987 sessions that amounted to 68% of all sessions of the application. The median average session duration per day in the Netherlands was 02:00 min (IQR 01:03–03:30) and the bounce rate was 3% which was the lowest of all countries (Table 1). The mobile application was most used on Monday, Tuesday, and Thursday, and the number of sessions was highest during the following time frames: 12–13 pm (9%), 17–18 pm (9%), and 13–14 pm (8%).

**Table 1:** List of top ten countries in which the application was used.

Country	New users	Sessions	Bounce rate (%)	Pages/session	Median session duration (min)
The Netherlands	1822	10,987	3	7,26	02:51

United States	1313	134,879	77	1.47	00:32

(not set)	964	982	87	1.24	00:18

United Kingdom	418	469	77	1.92	00:25

China	219	224	82	1.16	00:38

Japan	149	151	85	1.17	00:22

Germany	142	169	62	2.25	00:46

Italy	116	143	34	3.96	00:40

Brazil	93	95	89	1.48	00:08

Russia	54	282	48	1.62	02:59

Trial information pages were visited 15,896 times and the median time on a trial page ranged from 9 s to 47 s. The most frequently visited study pages are depicted in Table 2.

**Table 2:** All available trials in the application.

DCCG trials	Trial ID	Page views: n (%)	Median time on page: Sec (IQR)
CAIRO5	NCT02162563	2923 (18)	47 (18–161)

ORCHESTRA	NCT01792934	2117 (13)	41 (10–136)

CAIRO4	NCT01606098	1926 (12)	27 (7–87)

CHARISMA	NTR4893	1591 (10)	21 (4–74)

COLOPEC	NCT02231086	1575 (10)	26 (14–71)

RAPIDO	NCT01558921	1125 (7)	21 (7–58)

ASPIRIN	NCT02301286	1106 (7)	15 (5–53)

PLCRC	NCT02070146	956 (6)	11 (5–41)

TESAR	NCT02371304	787 (5)	19 (5–34)

FIT	NCT02243735	709 (4)	9 (4–22)

SALTO	NCT01918852	598 (3)	8 (3–22)

MRI2	NCT01721785	483 (3)	8 (3–22)

CONSTRUCT	NTR4673	321 (2)	9 (6–28)

Total		15896	

### 3.1. Online survey

**Figure 3:**
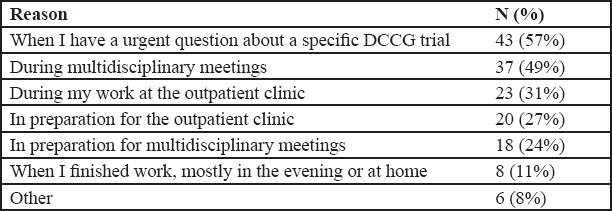
Overview of the reasons for application use. 75 of 121 people answered this question.

Of a total of 121 respondents, 76 (63%) answered that they have used the application, whereas 45 (37%) did not. Reasons for not using the application were unawareness of its existence (n = 26, 59%), preference for other resources (n = 8, 18%), or that the responder is not provided a smartphone at work (n = 4, 9%) (Figure 3).

Respondents were either medical doctors (n = 36, 47%), nurses (n = 19, 25%), researchers (n = 7, 9%), or data manager (n = 6, 8%), while 8 (11%) of the respondents defined their job as “other.” Respondents were working either in a teaching (40%), academic hospital (32%), or general hospital (19%), while 9% of the responders defined their working place as “other.” 83% of all respondents rated the application 4 or higher for satisfaction on a 5-point scale, and 86% recommended the application to a colleague. Highest reported reasons of use were urgent trial inquiry (57%) and usage during multidisciplinary meetings (49%).

## 4. Discussion

The present study illustrates that a smartphone application with concise information on multicenter trials on colorectal carcinoma is often used and that reported satisfaction with the application is high. More than 70% of the users are medical doctors or nurses, and the application is mostly used during multidisciplinary meetings and during work in the outpatient clinic. The DCCG Trials application was launched as an experimental tool that provides concise information on clinical trials which might benefit patient inclusion. This application also offers insights for implementation in other clinical fields.

Since the DCCG is based in the Netherlands, it was to be expected that most users derive from the Netherlands. The increased Dutch user duration rate supports the suggestion that the application is a useful tool when professionals encounter potential inclusions. The survey illustrated that the majority of the users were physicians in teaching hospitals [14]. Compared to academic centers, these hospitals often have a higher patient turnover but lower awareness of ongoing trials, which could be a potential cause of slow patient enrollment. The application directly offers information on inclusion and exclusion criteria, increasing the chances of patient inclusion. In addition, patient inclusion often has a certain momentum in clinical practice. If eligibility can directly be assessed by use of the application or when a potential trial candidate can directly be discussed with the trial coordinator, likelihood of patient enrollment increases. Finally, the option to directly update users with new trial information reminds users of the existence of a trial, indirectly increasing the likelihood of including patients in trials.

This is the first study presenting data of an application with the goal to improve clinical trial conduction and patient recruitment. Many applications have been developed in which trial information can be found [15-18]. However, no data are available about the effect of these applications on the conduction and recruitment of clinical trials. Little evidence is available for any intervention on the effect of patient recruitment in clinical trials [10]. It is difficult to prove the effect of the DCCG Trials application on trial enrollment. However, the fact that the application is frequently used by relevant users suggests that an application with relevant information on ongoing trials could have a positive impact on trial enrollment.

The current analyses have several limitations. First, presenting data on the duration of sessions and trial page visits does not necessarily reflect a positive user experience or positive effect on the conduction of a particular trial. Preferably, the effect of the application on inclusion rates is investigated, but the available data and trials were limited and a direct causal effect of the application on inclusion rates would be difficult to assess. Second, the survey was made available online, and respondents were recruited through the app, DCCG newsletter, and DCCG mailing list, which could have introduced a selection bias. It can be assumed that users that are very positive about the application are more willing to submit a survey, yet it could also be assumed that users with a very negative user experience are motivated to express their opinion.

Currently, the application is not available on iOS devices due to new technical requirements which are not met by the current version. The application is still available on Android devices. All DCGG studies from the application can be found in the trial app application which is available on all devices.

## 5. Conclusion

We report that our smartphone application with study information of ongoing colorectal cancer trials is frequently used and the majority of users is satisfied. The application provides easy to access and up-to-date information on ongoing clinical trials and is potentially useful to medical professionals in their busy daily practices. These results warrant the development of an application including all registered clinical trials in which users can select their own trials of interest, including all diseases and specialties.

## Conflict of Interest

The authors have no competing interest to disclose.

## Appendix

**Appendix 1:** An overview of the conducted survey.

Questions	Answer
1. Do you use the DCCG trials app?*	Yes/No

2. Why do not you use the DCCG Trials application?	Free text

3. What is your profession?	a. Medical doctor
	b. Research Nurse
	c. Researcher
	d. Data manager
	e. Other

4. What is your age?	n (years)

5. In what type of institution do you work?	a. Academic hospital
	b. Teaching hospital (STZ Ziekenhuis)
	c. General hospital
	d. Other

6. Are you satisfied with the DCCG Trials App	Score: 1–5 (Strongly disagree–strongly agree)

7. Do you agree with the following statement: I would recommend the DCCG Trials App to my colleagues.	Score: 1–5 (Strongly disagree–strongly agree)

8. When do you use the DCCG Trials application? (multiple answers possible)	a. During multidisciplinary meetings.
	b. In preparation for multidisciplinary meetings.
	c. During my work at the outpatient clinic.
	d. In preparation for the outpatient clinic.
	e. When I finished work, mostly in the evening or at home.
	f. When I have a (urgent) question about a specific DCCG trial.
	g. Other

9. For how long have you used the DCCG Trials App?	n (months)

10. What is your average session duration when you use the DCCG Trials App?	a. <2 min
	b. 2–5 min
	c. >5 min

11. Do you have any feedback that you would like to share?	Free text

* If answer is “No” only question 2 and 11 could be submitted, if answer is “Yes” question 2 could not be submitted, DCCG: Dutch Colorectal Cancer Group
